# Speciation of Chromium in Alkaline Soil Extracts by an Ion-Pair Reversed Phase HPLC-ICP MS Method

**DOI:** 10.3390/molecules24061172

**Published:** 2019-03-25

**Authors:** Barbara Leśniewska, Beata Godlewska-Żyłkiewicz

**Affiliations:** Institute of Chemistry, University of Bialystok, K. Ciołkowskiego 1K, 15-245 Bialystok, Poland; bgodlew@uwb.edu.pl

**Keywords:** ultrasound-assisted extraction, ion pair reversed phase HPLC coupled with inductively coupled plasma mass spectrometry, ionic chromium species, neutral and non-polar chromium fractions, alkaline soil extract

## Abstract

The aim of this work was to study by a hyphenated HPLC-ICP MS technique the chromium species released during alkaline extraction of various soils collected from a contaminated area of an old tannery. An ultrasound-assisted extraction procedure using 0.1 mol L^−1^ Na_2_CO_3_ solution was developed for the release of chromium species from the soil. The chromium species in the soil extracts were separated on a C_8_ column using EDTA and TBAH solution as a mobile phase. The use of an ICP-QQQ MS spectrometer in tandem mass configuration (MS/MS) combined with an octopole reaction system (ORS^3^) pressurized with helium allows one to eliminate spectral interferences during Cr determination in the soil extracts. The detection limit of the procedure was 0.08 µg L^−1^ for Cr(III) and 0.09 µg L^−1^ for Cr(VI) species. The trueness of the IP RP HPLC-ICP MS method was proved by an analysis of CRM 041 and CRM 060. The advantage of the proposed method is the analysis of soil extracts without their preliminary neutralization, which limits the losses of Cr(VI) due to the reduction process. The analysed soils mainly contained chromium in immobile forms (94.6–98.5% of the total Cr content). In all alkaline soil extracts mostly the Cr(VI) form was found, but in the extract of organic soils Cr(III) was also present. This arose from the reduction of Cr(VI) species by organic matter (humic acids) and Fe(II). The amount of formed Cr(III) species was dependent on the type of soil (content of organic matter, Mn and Fe) and its moistness. For the first time, the presence of neutral and non-polar chromium fractions in the soil extracts was also demonstrated. It was found that reliable speciation analysis results could be obtained for mineral soils.

## 1. Introduction

Chromium is widely used in various branches of industry, such as leather tanning, the iron and steel industry, metal finishing industry, petroleum refining, textile manufacturing and chemical production [1,2,3]. As a consequence, huge amounts of gaseous, liquid and solid waste containing chromium compounds are discharged into air, surface water, and soil, causing their contamination. Moreover, products manufactured with the use of chromium-containing pigments, may also be contaminated with this metal, e.g., textiles and toys [1,4]. Chromium present in contaminated soil can be taken up by plants and passed through the trophic chain to humans, or it can permeate into ground water. However, the trivalent and hexavalent forms of chromium differ significantly in terms of mobility and toxicity [5]. Chromium(III) is considered essential for the proper functioning of living organisms, whereas chromium(VI) is classified by the International Agency for Research on Cancer as a human carcinogen [6]. Therefore, the monitoring of the presence of hexavalent chromium in the soil is very important, especially in terms of human health and safety.

Generally, the Cr(III) species in the soil are considered as labile, where the HCrO_4_^−^ and CrO_4_^2−^ ions are the most mobile chromium forms [1,2,3]. The distribution of Cr(III) and Cr(VI) species in the soil depends on many factors, such as chromium concentration, soil properties (aeration, pH, organic matter, Fe(II) and Mn(IV) content, availability of electron donors/acceptors, redox potential, microbial activity), hydrolysis, redox and complexation reactions, as well as adsorption/desorption processes on soil particles or charged surfaces of macromolecular compounds (i.e., clay, humic acids or Mn(II) oxides) [1,2,3]. Another important parameter is the moistness of the soil. Moist soil is in partial equilibrium with atmospheric oxygen and in such conditions the oxidation and reduction of chromium species can occur simultaneously [7].

The determination of chromium species in soil is still one of the most challenging analytical tasks. Its difficulty results from the complexity of the analytical procedure, the risk of species’ interconversion at each step of the analytical procedure and the multiplicity of interactions of chromium with soil components [8]. Soil as a sample is also a demanding matrix due to its diversity, variability and complexity. The analytical procedures used for speciation analysis consists of five important steps, i.e., sampling, sample storage, pre-treatment (extraction, matrix separation), separation of species and their determination. The first four steps are the most crucial, because during these operations the interconversion of chromium species may occur. Therefore each step of the procedure for speciation analysis has to be optimized with respect to others and to the type of the sample.

For the speciation study of metals in contaminated soils various sequential extraction procedures based on the BCR protocol [9,10] or Tessier method [11] were often used. However, in the case of chromium most analytical procedures are focused on the selective extraction and determination of Cr(VI) in the soil, due to its high toxicity and mobility. Various extraction procedures have been proposed to release Cr(VI) from soils [12,13,14,15]. Exchangeable (mobile) forms of Cr(VI) were extracted with KH_2_PO_4_-K_2_HPO_4_ and K_2_HPO_4_ buffers [13,15,16]. Soluble (Na_2_CrO_4_) and sparingly soluble chromates (CaCrO_4_, BaCrO_4_, PbCrO_4_) were most often extracted with alkaline media, such as Na_2_CO_3_ [17], Na_2_CO_3_-NaOH solutions [18], Na_2_CO_3_ and NaOH with MgCl_2_ and phosphate buffer (EPA method 3060A) [19,20,21,22]. Some chelating agents (EDTA, DPTA) [12,23,24] were also proposed for Cr(VI) extraction. However, as was demonstrated in our previous work [25], these extraction procedures were not always selective for Cr(VI). The experiment performed on soils spiked with different chromium compounds has shown that the Cr(III) species are partially extracted with Na_2_CO_3_. As some authors have reported [19,26], partial oxidation of soluble Cr(III) to Cr(VI) make it unclear if these species were extracted under the experimental conditions, or were they firstly oxidised to Cr(VI) by soil components and then extracted into alkaline medium. Therefore, the aim of this work was to study chromium species released from various soils during their alkaline extraction.

So far for chromium speciation analysis in various extracts of soil a combination of ion chromatography (IC) and reversed phase high performance liquid chromatography (RP HPLC) with element-specific detection such as electrothermal atomic absorption spectrometry (ETAAS) and inductively coupled plasma mass spectrometry (ICP MS) was proposed [14,15,16,22,27,28,29]. The anion-exchange fast protein liquid chromatography with ETAAS [11] or ICP MS [16] detection was used for the determination of exchangeable Cr(VI) in the soil. In this method, quantitative elution of Cr(VI) species from a column was obtained in the wide range of sample pH values (3–13) using water and NaCl solution in gradient mode, while positively charged Cr(III) forms were eluted with the solvent front at pH 4. At higher pH (pH > 8) the signal of Cr(III) was not observed, as under such conditions Cr(III) exists mainly as Cr(OH)_3_ which is strongly adsorbed on the chromatographic column [16]. The separation of chromium species was also performed by reversed phase chromatography (RP) on a C_8_ column using mobile phase containing tetrabutylammonium phosphate (TBAP) [27] or tetrabutylammonium hydroxide (TBAH) [28,29] in 5% methanol as ion-paring reagent. For the simultaneous separation of Cr(III) and Cr(VI) species on IC and RP columns the formation of negatively charged complexes of Cr(III) e.g., with EDTA was necessary before sample injection [22,27,28,29]. However, the exhaustive elucidation of chromium speciation in soil extracts is still necessary.

In this work, the study of chromium speciation in alkaline extracts of soils collected from a contaminated industrial area was performed by a hyphenated HPLC-ICP MS technique. Based on our earlier experience [25], Na_2_CO_3_ was chosen for the extraction of soluble and sparingly soluble Cr(VI) forms from soil samples. In order to shorten the duration of the extraction, an ultrasound-assisted extraction procedure was chosen. The ion pairing reversed phase HPLC (IP RP HPLC) method [28] has been accordingly modified for separation of chromium species in alkaline soil extracts. The ICP-MS (QQQ MS) technique was used for determination of chromium and the efficiency of elimination of interferences by using collision/reaction cell pressurized with helium and oxygen was compared. To identify various fractions of chromium species present in the alkaline extracts and to examine the effect of soil components on distribution of chromium species a series of experiments were carried out. In order to provide the best fit of the method to the analysis of natural soils, various contaminated soils were used during the optimization separation and determination conditions of chromium species. The trueness of the developed method was confirmed by the analysis of certified soil reference materials (CRM 041 and CRM 060) with Cr(VI) reference value.

## 2. Results and Discussion

### 2.1. Optimization of Ultrasound Assisted Extraction Procedure of Chromium Species from Soil

The most beneficial effect of ultrasound in a chemical analysis is the acceleration of many physicochemical processes, such as dissolution, decomposition or leaching [30]. Different ultrasound sources such as an ultrasonic bath and probe have been used to accelerate the release of elements from the solid phase [30,31,32]. For example, during the extraction of water-, acid-soluble, and exchangeable fraction of Cd, Pb and Ni in soil the procedure was shortened from 16 h to 7 min by means of an ultrasound probe [9]. As the previously proposed procedure [25] for alkaline extraction of chromium species from soils (with 0.1 mol·L^−1^ Na_2_CO_3_) was time-consuming (rotation of soil suspension for 16 h) we decided to use ultrasounds to speed it up.

**Figure 1:**
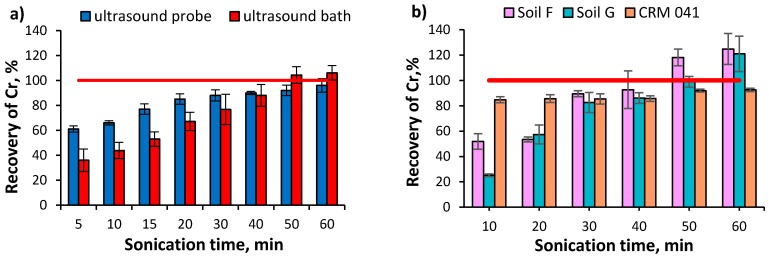
The influence of the sonication time on the recovery of Cr in alkaline extract of (**a**) organic soil spiked with BaCrO_4_ obtained by an ultrasound-assisted procedure using ultrasound probe (power: 26 W) and ultrasound bath (power: 100%); (**b**) natural organic soil (F,G) and CRM 041 obtained by an ultrasound assisted procedure using ultrasound bath (power: 100%) in comparison to results obtained by a classical procedure or certified value.

The organic soil spiked with BaCrO_4_ (an insoluble compound) was used for the optimization of the extraction procedure. Both a bath and a probe were used as the sources of ultrasound. The recovery of Cr was calculated against the spiked amount. In order to avoid interconversion of chromium species during the sonication process, a constant temperature (T = 25 ± 5 °C) was maintained. The ultrasound probe system was cooled down with flowing tap water [10], whereas the water in the bath was changed every 10–15 min. The influence of the power of the ultrasound probe and sonication time on the recovery of Cr from soil was studied. It was found that an increase of the probe’s power from 15 W to 26 W (for 5 min of sonication time) caused an increase of the recovery of Cr only from 54.0 ± 2.6% to 61.4 ± 2.6% (*n* = 3). Therefore, the maximum power (26 W) was applied during the optimization of the sonication time. The recovery of Cr increased along with the sonication time from 5 to 60 min and reached 92.0 ± 4.2 and 93.8 ± 4.5% (*n* = 3) for 50 min and 60 min of sonication, respectively (Figure 1a). The effect of the sonication time in an ultrasound bath was tested at 100% ultrasound power setting. A quantitative recovery of Cr was obtained for 50 min (104.4 ± 6.7%, *n* = 3) and 60 min (106.1 ± 5.8%, *n* = 3) sonication of the soil in the ultrasound bath. The recovery was not affected by the position of the sample in the bath (RSD < 8%). Therefore, regardless of the ultrasound source used, a 50 min sonication time was necessary for the quantitative recovery of chromium spikes. Since the use of an ultrasonic bath allows for simultaneous extraction of several samples at once, it was chosen for further research. The suitability of the ultrasonic bath for the acceleration of chromium extraction was tested for various types of soil (F and G) and CRM 041. The results obtained by an ultrasound-assisted procedure were always compared with the results obtained by a previously developed procedure [25]. Figure 1b shows the influence of the sonication time on the recovery of Cr from soils. Also for these samples the recovery of the analyte increased with time up to 93% after 40 min and 104–118% after 50 min of sonication. For a longer time of sonication (60 min), the recovery of the analyte was higher than 120%. The recovery of Cr from CRM 041 was at the level of 85–93% for sonication times from 10 to 60 min. In conclusion, for the extraction of chromium from the collected soil samples, the procedure using an ultrasound bath working at maximum power (100%) and 50 min of sonication time was applied. Such extracts were then analyzed for chromium content by ICP MS.

### 2.2. Optimization of the ICP MS Determination of Chromium

The determination of chromium species by the ICP MS technique is carried out using the most abundant ^50^Cr (4.4%), ^52^Cr (83.8%) and ^53^Cr (9.5%) isotopes [14,16,20,21,22,27,28,29]. Soil extracts, mobile phase and extraction solution contain large amount of compounds containing carbon, which can cause spectral interference during its determination [3,14]. The most common polyatomic interferences are caused by ^40^Ar^12^C^+^, ^35^Cl^16^O^1^H^+^, ^35^Cl^17^O^+^, ^34^S^18^O^+^ and ^36^S^16^O^+^ for ^52^Cr^+^ measurements and ^40^Ar^13^C^+^, ^40^Ar^12^C^1^H^+^, ^37^Cl^16^O^+^, ^35^Cl^17^O^1^H^+^, ^35^Cl^18^O^+^ and ^36^S^17^O^+^ for ^53^Cr^+^ measurements. For elimination of spectral interferences during measurements of chromium the ICP MS spectrometers equipped with a collision/reaction cell pressurized with helium [22], ammonia [27,28] or nitrogen [29] have been used.

In this work, in order to overcome the possible interferences, the use of the octopole reaction system (ORS^3^) pressurized with helium as collision gas or oxygen as reaction gas was tested. The tandem mass configuration of ICP MS/MS was applied in these studies. In the MS/MS mode the first quadrupole mass analyser (Q1), located in front of the ORS^3^ cell, operates as a mass filter, allowing only the target analyte mass to enter the cell and rejecting all other masses. In the collision/reaction cell the spectral interferences should be efficiently overcome, and the analyte ions go into a second quadrupole mass analyser (Q2), where they are measured. During the chromium determination with ORS^3^ pressurized with helium gas, Q1 and Q2 were setting at *m*/*z* 52, whereas when O_2_ gas was used Q2 was setting at *m*/*z* 68 and ^52^Cr^16^O^+^ ion was monitored. It is necessary to mention, that various tune modes with He, O_2_ or NH_3_/He gas may be used in the same measuring cycle, but in the case of the speciation analysis by HPLC-ICP MS method it is necessary to select only one set of collision/reaction cell parameters. In our study the gas flow rate was optimized for each gas that was used in order to obtain a high intensity for Cr standards with a low background level. The optimization was performed using a blank solution (1 mmol L^−1^ TBAH with 0.6 mmol L^−1^ EDTA or diluted carbonate solution) and standard solution of Cr at a concentration of 10 µg L^−1^. The optimal gas flow rate was 4 mL min^−1^ for He and 0.4 mL min^−1^ for O_2_. It was observed that using helium as a collision gas and oxygen as a reaction gas decreased the intensities of blank by 150-times and 400-times, respectively, in comparison to measurements performed in the standard mode (without using the ORS^3^ cell) due to the elimination of spectral interferences from ^12^C^40^Ar^+^ ions. In the case of Cr standard, a greater reduction of signal in the presence of oxygen than in the presence of helium was observed (20-times *vs*. 10-times), probably due to the low efficiency of conversion of ^52^Cr^+^ to ^52^Cr^16^O^+^ ion [33]. Therefore, helium was selected for the removal of spectral interference during the chromatographic separation of chromium species. For direct measurements of Cr by ICP MS both gases may be used in ORS^3^ cell, although more sensitive measurements were obtained when using helium. The detailed HPLC and ICP MS measurement conditions are listed in Table 1. The analysis of certified reference material CRM 041 under the selected conditions showed good agreement of certified values (86.3 ± 2.96 µg g^−1^) with obtained results (86.3 ± 2.98 µg g^−1^ for He and 79.9 ± 1.4 µg g^−1^ for O_2_, *n* = 3). It proved that both gases could efficiently eliminate spectral interferences of carbon/carbonate origin.

### 2.3. Optimization of the IP RP-HPLC-ICP MS Procedure

**Figure 2:**
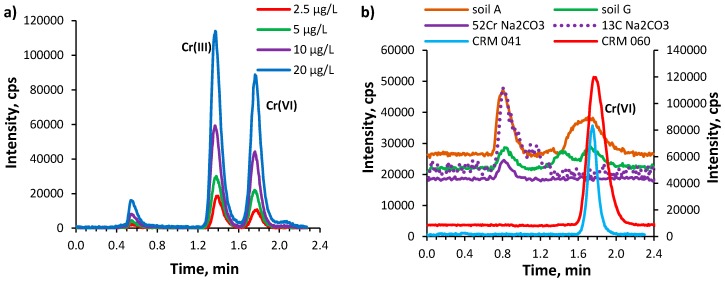
(**a**) Typical chromatogram for Cr(III) and Cr(VI) standards in diluted Na_2_CO_3_ obtained using optimized HPLC-ICP MS procedure (see Table 1), recorded at *m*/*z* 52; (**b**) Chromatogram for chromium species in alkaline extracts of mineral soil A (4-fold diluted), organic soil G (20-fold diluted) and certified reference materials CRM 041 (100-fold diluted) and CRM 060 (400-fold diluted), recorded at *m*/*z* 52. Chromatogram for Na_2_CO_3_ (20-fold diluted) was recorded at *m*/*z* 52 for chromium and at *m*/*z* 13 for carbon (dotted line). Additional right axis for CRM 041 and CRM 060.

In this work, the ion-pair reversed phase HPLC-ICP-MS method [28] was modified to study the speciation of chromium in alkaline extracts of various soils and CRMs. For the separation the Cr(III) cations are converted into negatively charged EDTA complexes, while positively charged TBAH is used to form ion-pairs with chromate (CrO_4_^2−^) and Cr(III)-EDTA^−^ anions. These species are retained and separated on the C_8_ column. Since the extraction method we applied was different from that used by Wolf et al. [28], the separation conditions, such as components of mobile phase, their concentrations and flow rate were examined and finally re-optimized in order to obtain a high sensitivity of measurements and good resolution of Cr(III) and Cr(VI) peaks. It was found that an increase of the TBAH concentration ranging from 0.4 mmol L^−1^ to 1.2 mmol L^−1^ resulted in an increase of retention times of both chromium species. Since it was reported [28] that the presence of methanol in the mobile phase helps to elute the compounds from the column, leading to narrower peaks and shorter eluting time, 5% methanol was added to the mobile phase. It was confirmed that methanol causes a decrease of retention times of chromium species by 6–8%. However, a higher content of organic carbon in the mobile phase also raised the background line (by about 40%) due to the occurrence of polyatomic interferences of ^40^Ar^12^C^+^ on *m*/*z* 52. For this reason methanol was eliminated from the mobile phase. An increase of the flow rate of the mobile phase from 0.4 mL min^−1^ to 1.0 mL min^−1^ caused a decrease of retention times of chromium species and a deterioration in the resolution of Cr(III) and Cr(VI) peaks. The best resolution of chromium species in standards and samples was obtained using 1 mmol L^−1^ TBAH and 0.6 mmol L^−1^ EDTA at pH 7.2 ± 0.1 as the mobile phase, passing through the column at a flow rate of 0.8 mL min^−1^. Under optimal conditions the characteristic peak for Cr(III) occurred at a retention time of 1.4 min, whereas for Cr(VI) at a retention time of 1.8 min (Figure 2a). The peak originating from a diluted extraction solution (Na_2_CO_3_) was observed in the dead volume of column (t_ret._ = 0.8 min).

It is well known that the kinetics of chelation of Cr(III) with EDTA are slow and the reaction is faster at higher temperatures. Therefore, we compared the peak areas of signals of Cr(III)-EDTA^−^ formed at room temperature within 60 min and during the course of heating at 60 °C for 30 min. Two different concentrations of Cr(III) standard were tested, namely 2.5 and 25 µg L^−1^. The differences between the signals of species formed at room and elevated temperatures were in the range of 1–4%. Therefore, in order to simplify the procedure the EDTA was added to all samples and left at room temperature for 60 min for complex formation. The optimal conditions for separation of chromium species in soil extract are presented in Table 1. During the analysis of soil extracts a shift in retention time and degradation of peak shape was observed after about 600 injections, which was also previously reported by Wolf et al. [28]. However, carrying out the separation procedure on a new column allowed us to obtain the same parameters for separation of chromium species.

### 2.4. Analytical Characteristic of the IP RP HPLC-ICP MS Method

The analytical performance of the proposed IP RP HPLC-ICP MS method was evaluated under the conditions given in Table 1. Calibration graphs for the quantification of Cr(III) and Cr(VI) species were obtained by plotting a peak area versus the concentration of the analytes. All calibration graphs were linear in the range of 1–100 μg·L^−1^ for Cr(III) and Cr(VI) ions with the correlation coefficient equal or higher than 0.999. The limit of detection (LOD), calculated as a signal-to-noise ratio of 3, was 0.080 μg L^−1^ (4 ng g^−1^) for Cr(III) and 0.090 μg L^−1^ (4.5 ng g^−1^) for Cr(VI), respectively. The reproducibility calculated from the peak area and retention time was satisfactory. The relative standard deviation was less than 3.5% (*n* = 6). The characteristics of the proposed method using the optimal chromatographic conditions are summarized in Table 2. The obtained parameters (LOD, LOQ, precision) were comparable to the previous ones reported in literature using a similar method [27,28]. In order to examine the influence of the soil matrix on Cr(VI) determination the standard addition method was applied. Chromium(VI) was added to extracts of mineral (soil C, D) and organic soils (soil F, G) and the procedure was carried out. It was found that calibration graphs obtained by the standard addition method were parallel to the graphs obtained by external calibration, which indicates that the effect of the soil matrix on the determination of Cr(VI) by the chromatographic method is negligible. The accuracy of the method was confirmed by an analysis of the reference materials of clay soil CRM 041 and CRM 060 with certified Cr(VI) content values. It was found that the determined contents of Cr(VI) in the reference materials were in good agreement with the certified value (Table 3).

### 2.5. Chromium Speciation Analysis in Soil Extracts by IP RP HPLC-ICP MS

The alkaline extracts of different types of soil were analyzed using the developed HPLC-ICP MS procedure. The description and characteristic of analyzed soils is given in Section 3.3 Materials and samples. In all analyzed extracts of soil samples and CRM materials the signals at a retention time of 1.8 min were registered, which indicates the presence of Cr(VI) (Figure 2b). The signal of Cr(III) at a retention time of 1.4 min was present in organic soils E, F, G. Based on the presence of ^13^C^+^ signal, carbon containing compounds, e.g., carbonates, were identified in the dead volume of the column. In order to identify whether the Cr(III) signal came from the original speciation of chromium in soil or from the transformation of chromium within extraction procedure or analysis, a series of experiments were performed.

Initially, the mass fraction of chromium extracted with Na_2_CO_3_ from soils was determined by ICP MS and HPLC-ICP MS methods (Table 3). The amount of Cr released from soils into the alkaline extract represented only 1.5–5.4% of its total content (determined after digestion of soil), which indicates that chromium in studied soils is present as an immobile form. These results are in accordance with general knowledge on the mobility of chromium in soil [1,2]. For analysis of CRMs, the good agreement of Cr(VI) content determined in alkaline extract by ICP-MS, HPLC-ICP MS and certified values was obtained. That proves that the used extraction procedure is effective for releasing of Cr(VI) and its determination by HPLC-ICP MS. However, it must be stressed that the matrix of CRM, which is a sandy clay soil with low content of organic matter and humic substances, could be simpler than the matrices of studied soils, so the extraction of Cr(VI) form CRMs was quantitative. The comparison of mass fraction of Cr determined by both methods, has showed that only 5–87% of the total amount of Cr in alkaline extract of soils was present in the column effluent. The mass fraction of Cr obtained by the chromatographic method was much lower for organic soil (soil E and G), that indicates the influence of the matrix of the soil on chromium species present in their carbonate extract. That discrepancy was not observed for well characterized and homogeneous CRMs.

The original speciation of chromium may change during the sample pretreatment step, as the extracted chromium species may interact with the soluble constituents of the soil and released solid particles. During the extraction with an alkaline solution of Na_2_CO_3_, soluble and insoluble Cr(VI) compounds are transformed into soluble chromate ions [18,34]. Some of the released species of Cr(VI) may be adsorbed on the surface of solid particles or undergo reduction to Cr(III) species in the presence of organic matter (i.e., humic or fulvic acids, humin), Fe(II) or S^2−^ ions. However, this last process is very unlikely under alkaline conditions (pH > 10) [1,2,3,7,21]. On the other hand, the majority of Cr(III) released from the soil precipitates as chromium carbonate or chromium hydroxide or undergoes adsorption on solid particles of the soil matrix. Insoluble, immobile and unreactive complexes of Cr(III) with organic ligands of high molecular mass, e.g., humic acids, can also be formed [18,35]. However, due to the formation of soluble complexes of Cr(III) with organic ligands of low molecular mass, e.g., citric, gallic, oxalic and fulvic acids [18], Cr(III) can be present in alkaline extract in some quantities. It can be oxidized to Cr(VI) only when an Mn(IV) oxide is present at a significant concentration [16]. This reaction is less realistic than the reduction of Cr(VI) to Cr(III) [3].

Considering these facts, the effect of the composition of alkaline extract of soils on the final chromium speciation was studied. First, the concentration of Mn in the alkaline extract of various soils was determined by ICP MS. It was found, that the calculated mass fractions of Mn (3–45 µg g^−1^) were lower than the concentration of MnO_2_ (100 µg g^−1^) which may induce the oxidation process of Cr(III) [16]. On the other hand, using carbonates for the extraction of soil favours the ageing of Cr(III) precipitate, which limits the oxidation of Cr(III) to Cr(VI) in the presence of oxidising agents [21,36]. Next, in order to check if chromium species could be changed by compounds released from the soil (e.g., organic matter), extracts of various mineral and organic soils were spiked with Cr(III) and Cr(VI) standards and submitted to the separation procedure. The quantitative recovery of Cr(III) and Cr(VI) spikes was obtained from all types of soil (Table 4), which indicates that the presence of liberated organic/inorganic compounds in the extract did not influence the reduction/oxidation processes of chromium species. This might be caused by the elimination of the neutralization step from the applied procedure which limits the risk of reduction of Cr(VI) to Cr(III) despite of the presence of reducing agents in soil extract. Analogous results were reported by Wolf et al. [28].

The conditions of the extraction process (type of soil, action of ultrasound, type of extractant) can also affect the original speciation. In order to study the potential chromium transformations in this step of the procedure the air-dry mineral and organic soils spiked with insoluble BaCrO_4_ (mineral: 43.1 ± 0.5 µg g^−1^ Cr, organic: 45.1 ± 1.2 µg g^−1^ Cr) were extracted with Na_2_CO_3_ under optimal conditions. Additionally, in order to simulate the environmental conditions, portions of the same soil samples were wetted with water to recover their natural moisture, next they were left for 24 h, and submitted to the extraction procedure.

**Figure 3:**
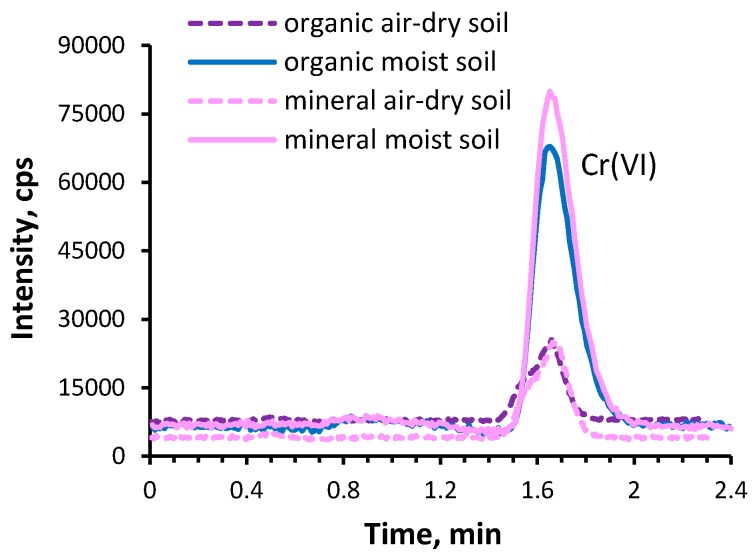
Chromatogram for chromium species in alkaline extracts of air-dry soil (100-fold diluted) and moist soil (50-fold diluted) spiked with insoluble BaCrO_4_, recorded at *m*/*z* 52.

The obtained extracts of mineral and organic soil varied in terms of colour. The dark colour of the organic soil extracts indicated the presence of humic substances in these solutions. The mass fractions of chromium extracted with Na_2_CO_3_ from the soils were determined directly and after chromatographic separation by the ICP MS method. As shown in Table 5, the results obtained by two methods are consistent, but the content of Cr determined in extracts is lower than the amount of Cr added to the soil. In all soil extracts only one peak at a retention time corresponding to the Cr(VI) form appeared in chromatograms (Figure 3). Therefore, it can be concluded that only the ionic form of Cr(VI) was present in all extracts.

Recoveries of Cr(VI) from mineral and organic soil samples were slightly different (Table 5). For air-dry mineral soil enriched with BaCrO_4_, the recovery of Cr(VI) was quantitative (99.3 ± 3.6, *n* = 3) as compared to the spiked Cr(VI) amount, while for moist soil the recovery was lower (78.2 ± 3.0%, *n* = 3). Probably, when the air-dry soil was wetted with water and extracted in an alkaline solution, iron (II/III)-bearing minerals were solubilized and were capable of reducing Cr(VI) to Cr(III). The capability of Fe(II) to reduce Cr(VI) in a solution over a pH range 2–10 and correlation between reduction of Cr(VI) and the presence of iron in extract of soil was reported by Guidotti et al. [37]. Hence, the formed Cr(III) was associated with Fe(III)-hydroxide forming Cr(III)-Fe(III)-precipitate, and removed from the extract during the filtration step. The losses of Cr(VI) from dry mineral soil, due to its less probable reduction by Fe(II), were lower.

Lower recovery of Cr(VI) from organic soil enriched with BaCrO_4_ was obtained for moist soil (52.3 ± 5.2%, *n* = 3) than for air-dry soil (70.0 ± 0.5%, *n* = 3). Bartlett et al. [7] reported that drying the soil causes the breakage of humic polymers of the soil into fragments and converts the soil’s organic matter into easily oxidized material. Therefore, when the soil is remoistened, Cr(VI) may be readily reduced to Cr(III). This process occurs more efficiently when air-dry soil was wetted with water before extraction with Na_2_CO_3_. Moreover, drying the soil alters the surface of manganese oxide and decreases its ability to oxidize Cr(III) [1,7]. Considering the reactions which could take place in the alkaline extract, the reduction of Cr(VI) to Cr(III) in the presence of humic substances, solubilised in an alkaline solution (pH about 9.5), is not excluded. Such a process may be facilitated in the presence of soluble Fe(III), which first can be reduced to Fe(II) by humic substances and then oxidized by Cr(VI) in a redox cycle [37]. The high content of Fe found in the extract of organic soil spiked with BaCrO_4_ (mass fraction of Fe = 5.7 ± 0.3 mg g^−1^, *n* = 3) indicates that Fe may induce the reduction of Cr(VI). Since no Cr(III) peak was identified in any of the chromatograms, probably the formed Cr(III), in the solid form, was separated from the solution during centrifugation or filtration, either as Cr(OH)_3_ (precipitated at pH 9.5) or together with particles of iron oxide/manganese oxide particles. The lower recovery of Cr(VI) released from soil, could be an effect of its adsorption on the solid particles of organic matter and in consequence its losses during the filtration of suspensions. However, the most probable process is the reduction of Cr(VI) to Cr(III) both in organic soil and in alkaline soil extract. It is worth noting, that such an interconversion of species was also observed in the alkaline soil extracts obtained using EPA method 3060A [20,21,22]. Such effects were not observed for chromatographic analysis of alkaline extracts of CRMs in this work, as the chromatograms display the presence only ionic form of Cr(VI) (Figure 2b). The risk of adverse reactions of the extracted Cr(VI) with constituents of CRMs was reduced due to the simpler matrix of these materials (sandy soil). In opposite to real samples, the obtained alkaline extracts of CRMs were colourless, clear (without the presence of suspended matter), with low concentrations of Mn and Fe. A more definite way to elucidate the processes of potential interconversions of chromium species in soil and alkaline extract is using of speciated isotope dilution SID-ICP-MS [3,14]. However, there access to this technique in chemical laboratories is still limited, and serious problems with the equilibration between sample species and spike species, as well as the proper preparation of spike solution still exists [3].

**Figure 4:**
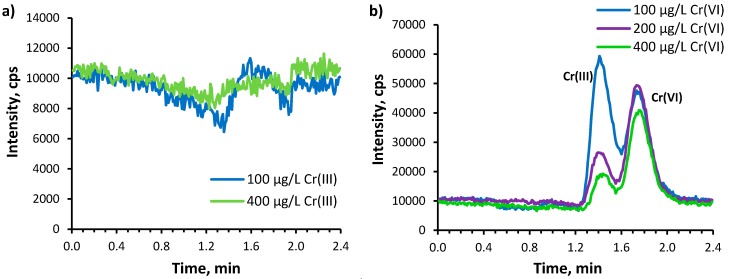
Chromatogram of (**a**) Cr(III) and (**b**) Cr(VI) standards at a concentration of 100–400 µg L^−1^ containing humic acids (0.7 g L^−1^) obtained after 24 h incubation at room temperature, recorded at *m*/*z* 52.

The difference in the mass fraction of chromium in soil extracted with Na_2_CO_3_ determined by the chromatographic and ICP MS methods was also observed (Table 3). It indicates the presence of chromium forms, which do not give any peak under the applied chromatographic conditions. As the proposed chromatographic method allows to separate only ionic forms of chromium, it was assumed, that neutral forms of chromium, which strongly retain on C_8_ column, are present in the soil extracts. The dark colour of the alkaline extracts of organic soils indicates that large amount of organic components of soils, e.g., humic and fulvic acids, were solubilised and passed into the solution. These humic substances may bind chromium into neutral complexes, which are strongly retained on the column. In order to study, which form of chromium, Cr(III) or Cr(VI) may form complexes, the humic acid at a concentration of 0.7 g L^−1^ was added to standards of Cr(III) and Cr(VI) at a concentration of 100–400 µg L^−1^ and left for 24 h at room temperature. Next, after adequate dilution, the solutions were submitted to the chromatographic separation procedure. On the chromatograms of Cr(III) standards with humic acids the signal of Cr(III) species was not present (Figure 4a). That means that Cr(III) was bound to the humic substance into form, which is strongly retained on the column. In the case of Cr(VI) standards, the peaks of Cr(VI) and Cr(III) forms were observed, which indicates the reduction of Cr(VI) to Cr(III) in the presence of humic substances (Figure 4b). It was found that more than 60–80% of Cr(VI) underwent reduction after 24 h at room temperature. However, the sum of the determined concentration of Cr(III) and Cr(VI) forms did not reach the initial concentration of Cr(VI) (Table 6). This means that a part of the formed Cr(III) species underwent transformation into forms strongly retaining on the RP column. The experiments done using the C_18_ column (as sample pretreatment step) showed that in alkaline extracts of mineral soil the fraction of non-polar chromium species (retained) was in the range 8–15%, while in extracts of organic soil from 20 to 35% of chromium was bound in non-polar fraction. The effluents from C18 column were submitted to the chromatographic separation procedure and determined. On the basis of the amount of chromium present in ionic forms and the mass balance, it was assessed that the fraction of chromium retained on the C_8_ column was in the range 50–75% for organic soil, while for mineral soil was negligible.

Therefore, we can conclude that chromium in mineral soil extracts is present mostly in ionic Cr(VI) form (85–92%) with a small amount of non-polar fraction (8–15%). In extracts of organic soil, apart from ionic Cr(VI) form (5–15%), the presence of neutral (50–75%) and non-polar (20–35%) Cr(III) complexes with humic acids was demonstrated. Other authors [27,28,29] have found only ionic Cr(VI) and Cr(III) forms in various soil extracts. The presence of other chromium forms in soil extract was not investigated. Good agreement of the sum of concentrations of ionic Cr(III) and Cr(VI) species with total concentration of chromium in the extracts was obtained in case of using HF (2%) in the mobile phase as extraction medium, that allowed liberating the chromium from cleavage of the metal-oxygen-silicone bond in soil [27].

## 3. Materials and Methods

### 3.1. Instrumentation

All RP-HPLC-ICP MS analyses were carried out using an Agilent 1260 Infinity HPLC system (Agilent Technologies, Waldbronn, Germany) connected to a Triple Quad ICP MS instrument (8800 ICP-QQQ, Agilent Technologies, Singapore, Singapore). The HPLC system was equipped with a G1311B quaternary pump with degasser (Agilent Technologies, Waldbronn, Germany) and a G1329B standard autosampler with a 100 μL polyetheretherketone (PEEK) sample loop (Agilent Technologies, Waldbronn, Germany). Chromium background originating from the metallic parts of the HPLC system was minimized by using a metal-free sample path of the LC bio-compatibility kit. The separation of chromium species was performed at room temperature on a reversed-phase C8 Brownlee cartridge column (Pecosphere, 3 µm diameter particles, 4.6 mm i.d. × 33 mm length) with a column holder (PerkinElmer, Machelen, Belgium). The outlet of the chromatographic column was directly connected to the nebuliser of the ICP MS instrument. A 8800 ICP-QQQ spectrometer fitted with MicroMist nebuliser, a Scott-type double pass spray chamber Peltier cooled and an octopole reaction system (ORS^3^) was used for the determination of chromium in the analysed samples. A nickel sampler and skimmer were used. Helium was used as a collision gas in ORS^3^ for the elimination of polyatomic interferences. The samples were delivered into plasma using an SPS4 autosampler in case of direct determination of chromium by ICP MS. Measurement conditions were optimized daily by short-term stability test runs in standard mode and with He-filled cell. The detailed HPLC and ICP MS measurement conditions are listed in Table 1. The data was processed using the Agilent Mass Hunter software.

An ultrasound processor, VCX 130 model (Sonics and Materials, Newtown, CT, USA) (max. power 130 W, max. frequency 20 kHz) equipped with a titanium probe and ultrasonic bath, Sonorex Digiplus DL 255H (Bandelin Electronics, Berlin, Germany) (frequency: 35 kHz, HF power: 160 W) were used for the extraction of soils, whereas an MPW-350 centrifuge (MPW Med. Instruments, Warsaw, Poland) was used for the separation of extracts from a suspension of soil.

An inoLab pH Level 1 (WTW, Weilheim, Germany) pH meter equipped with a SenTix 21 electrode (WTW) was used for the pH measurements.

### 3.2. Reagents

Stock solutions (20 g L^−1^) of Cr(III) as CrCl_3_ (Merck, Darmstadt, Germany) and (1.001 g L^−1^) of Cr(VI) as K_2_Cr_2_O_7_ (Sigma Aldrich, Steinheim, Germany) were used. Working standard solutions of chromium were prepared daily by appropriate dilution of the stock standards. A mobile phase was prepared by dissolving an appropriate amount of ethylenediaminetetraacetic acid dipotassium salt dehydrate (EDTA, Sigma Aldrich, Steinheim, Germany) in deionized water and by adding tetrabutylammonium hydroxide (TBAH, 1.0 mol L^−1^ solution in methanol, Sigma-Aldrich, Steinheim, Germany). The pH of the mobile phase was adjusted to pH 7.2 ± 0.1 with diluted HNO_3_ or NH_3_aq solutions (1:10, *v*/*v*). Nitric acid (67–69%, TraceSelect, Fluka, Seelze, Germany) was used for the digestion of soil samples. All standards and solutions used in the chromatographic method were prepared in the mobile phase. Deionised water was obtained from the Direct-Q3 water purification system (Millipore, Moisheim, France). For the extraction of chromium from the soil, a 0.1 mol L^−1^ solution of Na_2_CO_3_ prepared from solid substance (pure p.a, POCh, Gliwice, Poland) was used.

### 3.3. Materials and Samples

Certified reference materials of sandy clay soil CRM 041 and clay soil CRM 060 (Sigma Aldrich, Germany) with certified Cr(VI) content (CRM 041: 86.30 ± 2.96 µg g^−1^, CRM 060: 195.00 ± 9.02 µg g^−1^) were used for the accuracy (trueness) studies.

Non-contaminated mineral and organic soil spiked with BaCrO_4_ (mineral soil: organic matter 2.8%, Cr content 43.1 ± 0.5 µg g^−1^, pH_KCl_ 5.4; organic soil: organic matter 18.1%, Cr content 45.1 ± 1.2 µg g^−1^, pH_KCl_ 6.1) prepared according to procedure described previously [25] were used in this study for procedure optimization.

The soil samples (A–G) were collected from industrially contaminated area of an old leather tannery in Krynki (Podlasie Province, Poland). The soil’s parameters are shown in Table 7. Analyses of the characteristics of the soil (type of soil, content of organic matter and pH) were carried out by an accredited laboratory routinely engaged in such analysis, using standard analytical methods. 

The pH of the collected soils was alkaline, with only soil G being neutral. On the basis of a granulometric analysis, the examined soils were classified as very light soil (soil A and E), light soil (soil F) and medium soil (soil B, C, D, G). Three samples were classified as organic soils (soil E, F, G), while the remaining ones were classified as mineral soils. The total contents of chromium, manganese and iron were determined by ICP MS after digestion of soils in a mixture of HNO_3_: HF (5:1) according to the procedure described previously [25]. The total content of Cr in the soils is given in Table 3.

### 3.4. Procedures

#### 3.4.1. Extraction Procedure

For the extraction of chromium species from soils and certified reference materials, CRM 041 and CRM 060, an alkaline extraction procedure, described previously [25], was modified. In short, a mass of 1 g sample was weighted in glass tube and a portion of 50 mL of 0.1 mol L^−1^ solution of Na_2_CO_3_ was added. The tubes were placed in an ultrasound bath operated at 100% power for 50 min. In order to keep a constant temperature during sonication (T = 25 ± 5 °C) the water in the bath was changed every 10–15 min. Next, the suspensions were centrifuged at 3000 rpm for 15 min. The collected extracts of soils and CRMs were filtered through a nylon syringe filter with a pore diameter of 0.45 µm (Agilent Technologies) in order to separate all suspended matter from the solutions. All extractions were carried out in triplicate.

#### 3.4.2. Direct Determination of Cr in Soil Extract by ICP MS

The filtered extracts of soils, CRM 041 and CRM 060 were appropriately diluted with a 2% HNO_3_ solution and the concentration of chromium was determined by ICP MS using He gas in ORS^3^ for the elimination of spectral interferences. All measurements were performed using ^115^In as the internal standard. The mass fraction of chromium was calculated on the basis of an external calibration graph prepared in the extraction solution (Na_2_CO_3_ in 2% HNO_3_).

#### 3.4.3. Determination of Chromium Species in Soil Extracts by IP RP-HPLC-ICP MS

The calibration standards of Cr(III) and Cr(VI) species in the range of concentrations 1–25 µg L^−1^ were prepared in glass vials in a mobile phase, 1 mmol L^−1^ TBAH with 0.6 mmol L^−1^ EDTA (pH 7.2 ± 0.1), and left at room temperature for 60 min for formation of Cr(III)-EDTA^−^ complex. The extracts of soils, CRM 041 and CRM 060 were appropriately diluted with a mobile phase in glass vials and treated in the same way as standards. A volume of 50 µL of standard or sample was injected on the C_8_ column and analysed by the HPLC-ICP MS method. The mass fractions of Cr(III) and Cr(VI) species in soil samples extracted with Na_2_CO_3_ were calculated from external calibration graphs of Cr(III) and Cr(VI) prepared in a mobile phase. All analyses were performed in at least two replicates.

## 4. Conclusions

In this work we studied chromium speciation in alkaline soil extracts by means of HPLC-ICP MS. The developed ultrasound assisted extraction procedure of chromium species from soil using 0.1 mol L^−1^ Na_2_CO_3_ solution allowed us to shorten the extraction time from 16 h to 50 min, in comparison to the previously proposed procedure [25]. Good recovery of Cr(VI) for CRM 041 demonstrated the usability of the developed procedure for efficient extraction of Cr(VI) from soil. The proposed procedure is simpler than normalized EPA 3060A in terms of the composition of the extraction medium and extraction conditions. The potential of ICP-QQQ MS spectrometer for the elimination of spectral interferences caused by the extraction solution, the mobile phase and the sample matrix, during chromium determination in the soil extract was demonstrated. The accurate results of the Cr determination in soils and CRMs were obtained using tandem mass configuration (MS/MS) combined with ORS^3^ pressurized with helium gas. The proposed method allows to separate Cr(III) and Cr(VI) within the analysis time of less than 3 min. The quantification of Cr(III) and Cr(VI) form was performed on the basis of external calibration graphs. The advantage of the proposed method is the analysis of soil extracts without their preliminary neutralization, which limits losses of Cr(VI) due to the reduction process. The trueness of IP RP HPLC-ICP MS method was proved by an analysis of CRMs (CRM 041 and CRM 060) with certified values of Cr(VI).

The mass fraction of Cr, extracted with alkaline solution, in soils collected from contaminated area of the old tannery was low in comparison to the total content of chromium (1.5–5.4%), indicating presence of chromium in immobile form.

The chromatographic analysis of alkaline extracts of these soils showed mostly the presence of the toxic Cr(VI) form. However, in some extracts of organic soils ionic Cr(III) form was also observed. The performed studies indicate that Cr(III) arose from reduction of Cr(VI) species by organic matter and Fe(II) in soil or during extraction step. The amount of formed Cr(III) species depends on the type of soil (content of organic matter, Mn and Fe) and its moistness, therefore the physicochemical characteristic of the soil may be helpful in the interpretation of the results obtained during speciation analysis of chromium in soil. Our studies showed that apart from Cr(VI) in ionic form, in extract of mineral soils there is also a minor amount of non-polar chromium fraction, strongly retained on a C_8_ column. In alkaline extract of organic soils, chromium is present as ionic fraction (Cr(VI) and Cr(III)), as well as polar and non-polar fractions containing neutral complexes of Cr with humic acids. Therefore, the proposed IP RP HPLC-ICP MS method may be recommended for speciation analysis of chromium in mineral soil. The more definitive way to demonstrate stability or interconversion of chromium species in organic soil during sample preparation would be the use of SID ICP-MS with single or double spiking approach [3,14]. On the other hand, a new CRMs with a matrix more closely matches with natural (organic and mineral) soils are necessary for accurate speciation analysis of chromium in soil.

## Figures and Tables

**Table 1:** Optimal operational conditions for ICP MS and HPLC-ICP MS analysis.

HPLC-ICP MS	
**HPLC**	
Column	C_8_ cartridge column (3 µm diameter particles, 4.6 mm × 33 mm)
Mobile phase	1 mmol L^−1^ TBAH and 0.6 mmol L^−1^ EDTA, pH 7.2 ± 0.1
Flow rate	0.8 mL min^−1^
Injection volume	50 µL
Elution program	isocratic
Total analysis time	2.4 min
**ICP MS**	
RF power	1550 W
Plasma gas flow rate	15 L min^−1^
Auxiliary gas flow rate	1 L min^−1^
Nebulizer gas flow rate	0.9 L min^−1^
Sampling depth	10 mm
Spray chamber temperature	2 °C
Collision gas	He ^a,b^, O_2_^a^
Collision gas flow rate	He ^a,b^: 4 mL min^−1^; O_2_^a^: 0.4 mL min^−1^
Octopole bias	−5V
Octopole RF	190 V
Discrimination energy	He: 5 V ^a,b^; O_2_:−7V^a^
Scan type	MS/MS ^a,b^
Integration time	0.1 s ^a^, 0.5 s ^b^
Monitored ion (*m*/*z*)	^52^Cr^+^ (He mode) ^a,b^, ^52^Cr^16^O^+^ (O_2_ mode) ^a^, ^13^C^+^ (He mode) ^b,^
Internal standard (*m*/*z*)	^115^In^+ a^

^a^—ICP MS, ^b^—HPLC-ICP MS.

**Table 2:** Analytical characteristic of ICP MS and IP RP-HPLC-ICP MS methods.

	Calibration Graph Equation	LOD, µg L^−^^1^	LOQ, µg L^−^^1^	Precision (RSD, %)	
**ICP MS**				1-25 µg L^−1^ standards (*n* = 3)	
He as collision gas					
Cr *	y = 0.0604x + 0.0074	0.027	0.081	1.1–3.2	
Cr **	y = 0.0618x + 0.0148	0.023	0.069	1.8–4.6	
O_2_ as reaction gas					
Cr *	y = 0.0088x + 0.0007	0.085	0.255	1.5–4.5	
Cr**	y = 0.0100x + 0.0023	0.083	0.250	1.8–4.4	
**HPLC-ICP MS**				10 µg L^−1^ Cr(III) and Cr(VI) (*n* = 6)	
He as collision gas				t_ret._	area of peak
Cr(III) **	y = 159 809x + 14 411	0.08	0.23	0.9	3.3
Cr(VI) **	y = 153 210x + 7 103	0.09	0.26	0.2	2.5

* in 0.01 mol L^−1^ Na_2_CO_3_ in 2% HNO_3_ with using ^115^ In^+^ as internal standard. ** in mobile phase.

**Table 3:** Mass fraction of chromium extracted with Na_2_CO_3_ from soil determined by ICP MS and IP RP HPLC-ICP MS methods (mean value ± standard deviation, *n* = 3).

Sample	Total Content Cr ± SD, µg g^−1^	Cr Mass Fraction ± SD, µg g^−1^, ICP MS	Cr in Total Content ± SD, %	Mass Fraction of Chromium, µg g^−1^ ± SD, HPLC-ICP MS		$*\;\frac{HPLC-ICP\;MS}{ICP\;MS}\pm \mathrm{SD},\;\%$
				Cr(VI)	Cr(III)	
CRM 041	** 86.3 ± 2.96	86.3 ± 2.98	100 ± 3.4	86.3 ± 2.7	-	100.0 ± 3.2
CRM 060	** 195 ± 9.02	178.6 ± 9.20	91.6 ± 4.7	191.3 ± 7.6		107.1 ± 4.2
Soil A	69 ± 5	1.1 ± 0.1	1.5 ± 0.1	0.9 ± 0.2	-	87.3 ± 7.6
Soil B	42 ± 3	1.7 ± 0.2	4.0 ± 0.5	0.7 ± 0.1		41.8 ± 2.1
Soil E	2336 ± 30	44.6 ± 5.9	1.9 ± 0.3	2.2 ± 0.4	0.2 ± 0.02	5.3 ± 0.9
Soil F	142 ± 6	2.1 ± 0.1	1.5 ± 0.07	0.42 ± 0.02	0.35 ± 0.05	36.3 ± 5.8
Soil G	284 ± 8	15.6 ± 1.5	5.4 ± 0.5	1.3 ± 0.7	0.9 ± 0.2	14.5 ± 3.5

* percentage of the sum of Cr species determined by HPLC-ICP MS to the total mass fraction of Cr determined by ICP MS. ** certified value of Cr(VI).

**Table 4:** The recovery of Cr(III) and Cr(VI) spikes from various type of soil determined by IP RP HPLC-ICP MS method.

Sample	Added Cr(VI), µg L^−1^	Recovery of Cr(VI) ± SD, %, *n* = 3
Soil C (M)	1.4 2.6	111.1 ± 2.1 115.4± 3.5
Soil E (O)	2.7 5.4	115.2 ± 3.1 113.6 ± 2.5
Soil F (O)	1.1 2.7	101.2 ± 2.4 104.4 ± 2.8
Soil G (O)	5 µg·L^−1^ Cr(VI) + * 5 µg·L^−1^ Cr(III)	104.8 ± 4.9 * 89.9 ± 12.8

O-organic soil, M-mineral soil. * concentration/recovery of Cr(III).

**Table 5:** The recovery and mass fraction of Cr(VI) extracted with Na_2_CO_3_ from soil spiked with BaCrO_4_ determined by ICP MS and IP RP HPLC-ICP MS methods (mean value ± standard deviation, *n* = 3).

Soil Spiked with BaCrO_4_	Added Cr, µg g^−1^	Mass Fraction of Cr, µg g^−1^		$*\frac{HPLC-ICP\;MS}{ICP\;MS},\;\%$	Recovery, %	
		ICP-MS	HPLC-ICP MS		ICP-MS	HPLC-ICP MS
Mineral: air-dry	43.1	40.2 ± 1.9	42.8 ± 1.5	106.5 ± 3.7	93.4 ± 4.5	99.3 ± 3.6
moist		33.7 ± 1.3	31.8 ± 0.4	94.4 ± 3.9	78.2 ± 3.0	78.2 ± 3.0
Organic: air-dry	45.1	32.8 ± 1.3	31.6 ± 0.2	96.3 ± 0.6	72.8 ± 2.9	70.0 ± 0.5
moist		24.9 ± 1.7	23.6 ± 2.4	94.8 ± 3.0	55.2 ± 3.7	52.3 ± 5.2

* percentage of the sum of Cr species determined by HPLC-ICP MS to the total mass fraction of Cr determined by ICP MS.

**Table 6:** Concentration and recovery of Cr(III) and Cr(VI) spike determined in standards with humic acid by HPLC-ICP MS method.

Humic Acid + Cr, µg·L^−^^1^	Determined Concentration, µg·L^−^^1^		Recovery of Cr, %
	Cr(III)	Cr(VI)	
Cr(III)			
100			0.27
200	0.72		0.35
400	1.44		0.36
Cr(VI)			
100	42.2	36.9	79.1
200	27.4	75.6	51.5
400	32.4	79.2	27.9

**Table 7:** The characteristics of soil samples collected from the contaminated area of an old leather tannery.

Sample	Organic Matter, %	pH_KCl_	Total Content of Mn ± SD, µg g^−1^, *n* = 3	Total Content of Fe ± SD, mg g^−1^, *n* = 3
Soil A (VL)	1.9	7.8	132 ± 9	13.9 ± 0.7
Soil B (M)	2.9	7.3	570 ± 31	10.6 ± 0.5
Soil C (M)	3.1	7.4	445 ± 11	8.6 ± 0.3
Soil D (M)	4.3	7.4	453 ± 17	8.6 ± 0.3
Soil E (VL)	5.4	7.7	218 ± 13	6.1 ± 0.3
Soil F (L)	7.3	7.3	268 ± 8	4.1 ± 0.3
Soil G (M)	10.1	7.2	348 ± 6	14.2 ± 1.8

VL—very light soil, L—light soil, M—medium soil.
